# Associations of language barriers with very preterm children’s behavioural and socio-emotional problems across Europe

**DOI:** 10.1038/s41390-024-03623-4

**Published:** 2024-11-24

**Authors:** Julia Jaekel, Adrien M. Aubert, Nils Jaekel, Raquel Costa, Samantha Johnson, Jennifer Zeitlin, the SHIPS Research group, J. Lebeer, P. Van Reempts, E. Bruneel, E. Cloet, A. Oostra, E. Ortibus, I. Sarrechia, K. Boerch, P. Pedersen, L. Toome, H. Varendi, M. Männamaa, P. Y. Ancel, A. Burguet, P. H. Jarreau, V. Pierrat, P. Truffert, R. F. Maier, M. Zemlin, B. Misselwitz, L. Wohlers, M. Cuttini, I. Croci, V. Carnielli, G. Ancora, G. Faldella, F. Ferrari, C. Koopman-Esseboom, J. Gadzinowski, J. Mazela, A. Montgomery, T. Pikuła, H. Barros, R. Costa, C. Rodrigues, U. Aden, E. S. Draper, A. Fenton, S. J. Johnson, S. Mader, N. Thiele, J. M. Pfeil, S. Petrou, S. W. Kim, L. Andronis, J. Zeitlin, A. M. Aubert, C. Bonnet, R. El Rafei, A. V. Seppänen

**Affiliations:** 1 Faculty of Education and Psychology, University of Oulu, Oulu, Finland; 2 Public Health Unit, Finnish Institute for Health and Welfare (THL), Helsinki, Finland; 3 Department of Psychology, University of Copenhagen, Copenhagen, Denmark; 4 Department of Health Sciences, University of Leicester, Leicester, UK; 5 Department of Psychology, University of Warwick, Warwick, UK; 6 Department of Paediatrics I, Neonatology, Paediatric Intensive Care, Paediatric Neurology, University Hospital Essen, University of Duisburg-Essen, Essen, Germany; 7 Université Paris Cité, Inserm, INRAE, Centre for Research in Epidemiology and StatisticS (CRESS), Obstetrical Perinatal and Pediatric Epidemiology Research Team, EPOPé, Paris, France; 8 Department of English, German and Romance Studies, University of Copenhagen, Copenhagen, Denmark; 9 EPIUnit, Instituto de Saúde Pública, Universidade do Porto, Porto, Portugal; 10 Laboratório para a Investigação Integrativa e Translacional em Saúde Populacional, Porto, Portugal; 11 Department of Family Medicine & Population Health (FAMPOP), Disability Studies, Faculty of Medicine & Health Sciences, University of Antwerp, Antwerp, Belgium; 12 Laboratory of Experimental Medicine and Pediatrics, Division of Neonatology, University of Antwerp, Antwerp, Belgium; 13 Study Centre for Perinatal Epidemiology Flanders, Brussels, Belgium; 14 Centre for Developmental Disabilities, Neonatal Intensive Care, Oost Limburg Hospital, Genk, Belgium; 15 Vrije Universiteit Brussel Faculteit Geneeskunde en Farmacie; Paediatric Neurology, Universitair Ziekenhuis Brussels, Jette, Belgium; 16 Centre for Developmental Disabilities, Ghent University Hospital, Ghent, Belgium; 17 Centre for Developmental Disabilities, Leuven University Hospital, Leuven, Belgium; 18 Department of Neuropediatrics, University of Leuven, Leuven, Belgium; 19 Department of Paediatrics, Hvidovre Hospital, Copenhagen University Hospital, Hvidovre, Denmark; 20 Department of Neonatology, Hvidovre Hospital, Hvidovre, Denmark; 21 Tallinn Children’s Hospital, Tallinn; University of Tartu, Tartu, Estonia; 22 University of Tartu, Tartu University Hospital, Tartu, Estonia; 23 University of Tartu, Department of Paediatrics, Institute of Clinical Medicine, Tartu, Estonia; 24 Division of Pediatrics 2, Hôpital du Bocage; INSERM CIE1, CHRU Dijon, Université de Dijon, Dijon, France; 25 Université Paris Descartes and Assistance Publique Hôpitaux de Paris, Hôpitaux Universitaire Paris Centre Site Cochin, DHU Risks in pregnancy, Service de Médecine et Réanimation néonatales de Port-Royal, Paris, France; 26 Department of neonatology, centre hospitalier intercommunal Créteil, Créteil, France; 27 Department of Neonatology, Jeanne de Flandre Hospital, Lille CHRU, Lille, France; 28 Children´s Hospital, University Hospital, Philipps University Marburg, Marburg, Germany; 29 University Medical Center, Homburg, Germany; 30 Institute of Quality Assurance Hesse, Eschborn, Germany; 31 Clinical Care and Management Innovation Research Area, Bambino Gesù Children’s Hospital, IRCCS, Rome, Italy; 32 Maternal and Child Health Institute, Marche University and Salesi Hospital, Ancona, Italy; 33 Neonatal Intensive Care Unit, Ospedale degli Infermi, Rimini, Italy; 34 Neonatal Intensive Care Unit, University Hospital S. Orsola-Malpighi, Bologna, Italy; 35 Department of Pediatrics and Neonatology, Modena University Hospital, Modena, Italy; 36 Department of Neonatology, Wilhelmina Children’s Hospital, Utrecht, the Netherlands; 37 Department of Neonatology, Poznan University of Medical Sciences, Poznan, Poland; 38 Department of Neonatology and Neonatal Infectious Diseases, Poznan University of Medical Sciences, Poznan, Poland; 39 EPIUnit-Institute of Public Health, University of Porto, Porto, Portugal; 40 Department of Women’s and Children’s Health, Karolinska Institutet, Stockholm, Sweden; 41 Department of Population Health Sciences, University of Leicester, Leicester, UK; 42 Newcastle University, Newcastle upon Tyne, Callaghan, Australia; 43 European Foundation for the Care of Newborn Infants (EFCNI), Munich, Germany; 44 Nuffield Department of Primary Care Health Sciences, University of Oxford, Oxford, UK; 45 Warwick Medical School, University of Warwick, Coventry, UK

## Abstract

**Background:**

Very preterm birth (<32 weeks gestation, VP), immigrant background, and language barriers are all independently associated with a high risk for mental health problems in childhood, but research has neglected the long-term development of immigrant children born VP. We assessed whether behavioural and socio-emotional problems of 5-year-old children born VP growing up across different language contexts in the European Union are associated with an immigrant background and linguistic distance of families’ mother tongue (L1) to the host countries’ official languages.

**Methods:**

Data are from a population-based cohort including all VP births in 2011/12 in 11 European countries; a total of 3,067 children were followed up at 2 and 5 years of age. Behavioural and socio-emotional difficulties were assessed using the parent-reported Strengths and Difficulties Questionnaire (SDQ).

**Results:**

Mixed-effects models showed that a larger linguistic distance of children’s L1 to the host countries’ official language was associated with higher SDQ total scores (0.02 [0.01, 0.03]), after adjusting for a wide range of social risks, biological, and perinatal clinical factors.

**Conclusion:**

Language barriers in the form of linguistic distance between VP children’s L1 and countries’ official languages play a critically important role for the behavioural and socio-emotional development of immigrant children born VP.

**Impact:**

- Immigrant children born very preterm across Europe face systemic inequalities such as language barriers. Language barriers can be operationalised as a continuous linguistic distance score between children’s mother tongues and countries’ official languages. Linguistic distance plays an important role for the behavioural and socio-emotional development of immigrant children born VP. Research, policy, and practice need to better account for language barriers to increase equity in health and education.

## Introduction

Every year more than 13 million babies (>10% of all births) are born preterm (<37 weeks of gestational age (GA)) worldwide.^1–3^ In Europe, 8.7% of all infants are born preterm, equating to 690 931 live births in 2014, while 1% are born very preterm (VP, <32 weeks of GA)0.^1^ With improvements in neonatal care, the survival rate of those at highest risk for developmental sequelae - infants born VP and extremely preterm (<28 weeks of GA) continues to increase. VP birth is associated with a cluster of mental health problems that include difficulties with attention regulation, emotions, and social behaviour.^4,5^ However, studies from different countries unequivocally show that the association of VP birth with long-term adverse outcomes varies according to environmental contexts such as family socio-economic status and ethnic background.^6–8^ An important but often neglected environmental factor is immigrant status, i.e., a child having one or two parents not born in the host country, due to having moved away from their country of origin.^9^

From 2000 to 2020, migration increased by 62%, now concerning over 281 million people.^10^ Europe accounted for the largest rise in immigrant populations worldwide,^11^ though the percentage of foreign-born residents varies substantially between countries. As a result of migration, linguistic diversity is growing exponentially worldwide. This is an important concern, as immigrants’ proficiency in their host country’s official majority language correlates with income,^12^ educational attainment,^13^ integration into society,^14^ health care utilisation,^15–17^ and overall health.^18,19^ Children of immigrants are at increased risk for language, cognitive, and mental health problems.^20–23^ For example, Turkish immigrant children in Germany are more likely to have behavioural and emotional problems than their native peers.^24^ However, immigrant children are often growing up with an intersectional accumulation of socio-economic vulnerabilities.^25^ Intersectionality refers to the understanding that everyone lives with their own unique identities and experiences of discrimination, and we must consider everything that can marginalise individuals, including language, gender, skin colour, education, sexual orientation, physical and mental ability, etc.^26,27^ For example, an immigrant girl from Syria born VP might experience cumulative biases and discrimination based on her language abilities, gender, family socio-economic background, outer appearance, as well as attention and emotional problems. The inequalities that children face can be further exacerbated by parents’ language difficulties that pose barriers to the use and quality of healthcare and education.^28–32^ For example, research shows that immigrant children’s high risk for behavioural and emotional problems is actually explained by their mothers’ language barriers and intersectionality of inequalities such as low education and unemployment.^24,28–30,32^ Host country official language skills constitute a critical resource for immigrant parents when it comes to navigating their new society,^12^ since language difficulties create misunderstandings, stigma, and discrimination. We hypothesise that higher levels of behavioural and emotional problems among preterm children growing up with language barriers are an indirect consequence of their own and their parents’ accumulated experiences of miscommunication. For example, immigrant mothers and fathers of children born preterm identify a range of challenges in their daily lives, including lack of understanding and feeling left alone with their children’s healthcare needs.^33^ Compromised communication due to language barriers can cascade into feelings of frustration and isolation, and subsequently increase the risk for behavioural and socio-emotional problems.

**Fig. 1:**
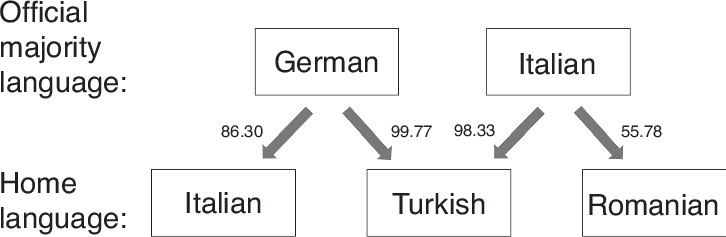
Linguistic distance scores for selected languages in the sample.

Moreover, most research on immigration uses categorical variables to define immigration status (e.g., immigrant vs. native). The comparison of ‘immigrant’ versus ‘native’ presumes that the former comprises a homogeneous group with similar linguistic traits as well as economic resources and cultural backgrounds and thereby neglects substantial variations between different languages and societal contexts that are associated with barriers to access to education, healthcare, and social participation.^34,35^ Speakers of other languages than their host countries’ official majority languages experience different levels of barriers, depending on the degree of similarity with their mother tongue (L1). For instance, some languages may be more or less mutually intelligible within their language families, such as Danish, Swedish, or Norwegian,^36^ making them relatively easily attainable for their speakers. Accordingly, some immigrants may have a potential advantage over others, based on linguistic similarities of their L1 to the host society’s official majority language(s). To better operationalize variations in the individual and contextual processes and conditions associated with immigrant health such as language barriers, we propose to assess the role of linguistic distance (LD) between a person’s L1 and the official language of the host country. LD here refers to the lexical and phonological level of similarity between two languages and is associated with proficiency development in the host country’s official language.^37,38^ Accordingly, LD can be understood as a language learnability index or as a fine-grained process factor that breaks up the binary immigration variable based on individual differences between languages.^35,37^ High LD, i.e., greater distance between two languages, has been associated with poor health,^34^ low educational attainment,^35,39^ and low income.^12,40^ In Germany, for instance, LD may indicate the level of similarities and differences between Turkish, Italian, and German. Turkish L1 children, for example, need to overcome a larger LD to the majority language German (LD = 99.77 points) than Italian L1 children in Germany (LD = 86.30 points), whereas in Italy, children speaking Romanian as their L1 have to overcome a fairly low LD of 55.78 points (Fig. 1). LD is associated with individuals’ abilities to draw on the linguistic resources available to them in their L1 and also suggests that individuals have different starting points in their learning of a second or foreign language. The learning process may be facilitated by reduced cognitive load for those with a lower LD, allowing them to focus on other less familiar linguistic properties involved in the new language.^35^

Accordingly, VP birth, immigrant status, and LD are all associated with high risk for mental health problems in childhood, which in turn have major implications for life-long developmental outcomes.^41–43^ Based on our recent proof-of-concept studies,^35,44^ we hypothesise that while language barriers in the form of LD are a global phenomenon affecting all children, exposure to the two different adversities of VP birth and language barriers in combination with high risk for other intersectional vulnerabilities such as low education and minority cultural identity creates multiplied risks for some children that need to be considered in the provision of screening and support services. Despite the evident importance of these early life risks and possible double jeopardies to development, very few studies have assessed associations of preterm birth and immigrant background with developmental outcomes beyond infancy.^45–47^ One study from the Netherlands found that multilingualism was associated with low cognitive and verbal outcomes of VP children at 2 and 5 years of age.^48^ Non-European born immigrant status was associated with increased risk for behavioural/psychosocial and multi-domain impairments at age 5.5 years.^49^ These studies provide important pointers, but they operationalised multilingual and immigrant status categorically, neglecting more fine-grained underlying process factors such as language barriers. Overall, societies in Europe are characterised by having high national incomes and universal insurance or health coverage, but nevertheless >2-fold disparities have been documented in risk-adjusted morbidity after VP birth between European regions.^50^ These variations in outcomes may be associated with differences in families’ heritage languages that create barriers for access to health care, education, and societal inclusion. Such barriers may have short- and long-term effects on VP children’s outcomes, but to our knowledge, such studies on fine-grained comparisons of family languages do not exist.

The aim of this study is to assess the mental health of children born VP with and without immigrant background who are growing up across different language contexts in the European Union. We tested the following hypotheses:

1. Immigrant status and LD of families’ L1 to the host countries’ official languages are both associated with behavioural and socio-emotional problems of 5-year-old children born VP.
2. LD is independently associated with behavioural and socio-emotional problems of children born VP, after adjusting for the effects of immigrant status, social risks as well as biological and perinatal clinical variables, and accounting for the nestedness of data within families (for multiple births) and countries.

## Methods

**Fig. 2:**
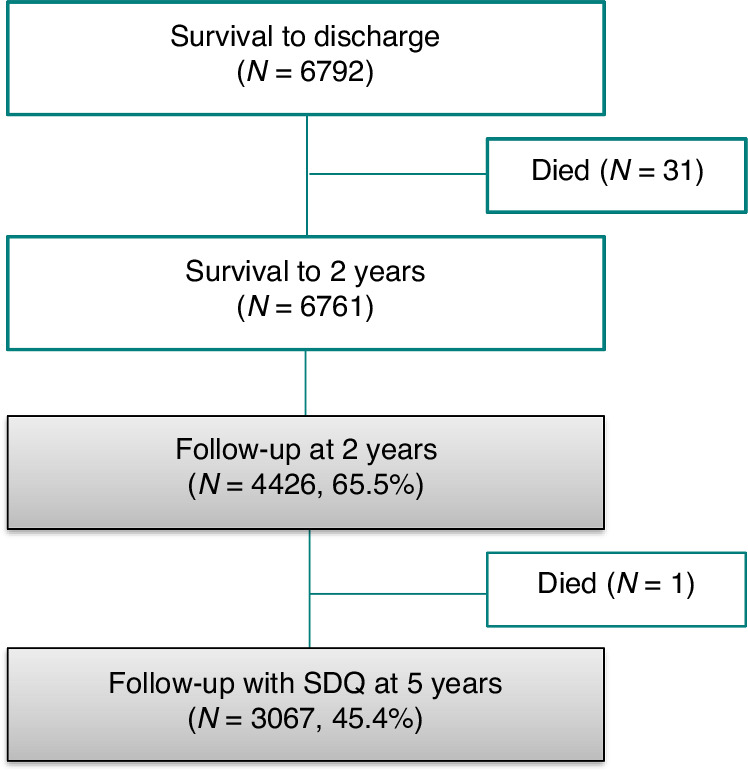
Study flow diagram from birth to 5 years of age.

We used data from the Effective Perinatal Intensive Care in Europe (EPICE) population-based cohort including all births between 22 + 0 and 31 + 6 weeks of GA in 2011/12 in 19 regions in 11 European countries, and with children followed-up to 5 years of age as part of the Screening to Improve Health in Very Preterm Infants in Europe (SHIPS) study.^51^ Data were collected from obstetric and neonatal records during neonatal hospitalization and from parental questionnaires at 2 years of corrected age and 5 years of chronological age. During the recruitment period, 7900 live-born VP infants were included, of whom 6,792 survived to discharge. As shown in Fig. 2, 6,761 children survived to 2 years of corrected age of which 65.5% were included in the 2-year follow-up. Of these, 3,067 (45.4% of all survivors) were included in the 5-year follow-up and had complete data on mental health.

### Procedures

Parental consent was obtained at both follow-up waves. Each country team received ethical authorisations from local regional or hospital ethics boards according to national legislation. The European study was approved by the French Advisory Committee on Use of Health Data in Medical Research (CCTIRS, for EPICE) and the French Expert Committee for Research, Studies and Evaluations in the field of Health (CEREES for SHIPS) as well as the French National Commission for Data Protection and Liberties (CNIL).

### Instruments

#### Children’s behavioural and socio-emotional problems

At 5 years, behavioural and socio-emotional difficulties were assessed using the parent-reported Strengths and Difficulties Questionnaire (SDQ), administered in each country’s official majority languages. The SDQ is a cross-culturally valid short screening instrument containing 25 items that load on five subscales measuring emotional problems, conduct problems, hyperactivity-inattention, peer relationship problems, and prosocial behaviour.^52^ Each item is scored on a 3-point Likert-type scale (0=not true, 1=somewhat true, 2=certainly true). A SDQ total score is obtained by summing the scores of four scales (prosocial behaviour not included; range 0–40, with some items reverse-coded) with a higher score indicating more problems.

#### Immigrant status and linguistic distance

Immigrant status was operationalised as a binary variable based on children’s mothers’ country of birth (born in the country vs. foreign-born). Children’s L1s were assessed via parent questionnaires at 2 years of age. This information was used to calculate the average LD to the host countries’ official language as one continuous variable (see Appendix 1 for a list of languages according to country). For 204 children, information on their families’ L1 was missing and mothers’ country of birth information was used instead to infer L1. LDs were operationalized using data from the Automated Similarity Judgement Program (ASJP)0.^53^ The LD calculated by the ASJP is based on 40 universally important, culturally independent everyday words and based on the normalized Levenshtein distance,^54^ i.e., the number of changes, including deletions, insertions, or substitutions, required to transform the phonetic representation of a word from one language to another. For example, fish (English) to Fisch (German) has a Levenshtein distance of 2, whereas fish (English) to balık (Turkish) has a Levenshtein distance of 5. For a detailed discussion of the statistical procedures employed in the calculation of the ASJP LD score please see.^55^ In the current study, LD was operationalised as one continuous score for each child.

#### Social risk

Information on mothers’ education (International Standard Classification of Education (ISCED) scores,^56^ binary coded into low (ISCED 0–2) vs. medium-high (ISCED 3–8)), single mother status (single vs. married or cohabiting), and household unemployment status (employed vs. at least one parent unemployed) was collected via questionnaires at age 2 and 5 years.

#### Biological and perinatal clinical variables

Information on child GA (weeks), biological sex (female vs. male), multiple birth status (singleton vs. multiple), parity (primiparous vs. multiparous), mother’s age at birth (years), bronchopulmonary dysplasia (BPD, with supplemental oxygen and/or ventilatory support (continuous positive airway pressure or mechanical ventilation) at 36 weeks of postmenstrual age, binary coded yes/no) and any severe neonatal morbidities (binary coded yes/no; defined as a composite measure of cystic periventricular leukomalacia, intraventricular haemorrhage grades III or IV, severe necrotizing enterocolitis requiring surgery or peritoneal drainage, or retinopathy of prematurity at least stage 3) was collected from medical records.

### Analysis strategy

Firstly, considering that participant attrition at 5 years was mainly associated with social disadvantage,^57^ unweighted regression models were compared with models using inverse probability weighting (IPW)0.^58^ As results were similar, IPW estimation was subsequently applied to all models. Participants with complete data at ages 2 and 5 were included in main analyses. Descriptive analyses and mixed-effects linear regressions were carried out in Stata version 17.0. First, to separately test the univariate associations of VP children’s LD and immigrant status with their behavioural and socio-emotional problems we ran two three-level models (i.e., level 1: individuals; level 2: families (i.e., siblings due to multiple births); level 3: countries), one including a fixed effect of children’s LDs from their L1 to the host country’s official language, the other including a fixed effect of mother’s country of birth, on SDQ total scores. Next, we entered both of these variables into one model to adjust their effects for each other. To adjust for social risks, we then added fixed effects of mothers’ education, single mother status, and household unemployment status. In the last step, we additionally added fixed effects of biological and perinatal clinical variables, i.e., child GA, sex, multiple birth, mothers’ age, parity, BPD, and severe neonatal morbidities. As part of a sensitivity analysis and to minimise risk of false interpretation due to distribution biases, the same models were repeated within the subgroup of children with foreign-born mothers.

## Results

Table 1 displays descriptive information for the cohort of children followed up at both 2 and 5 years comparing native (*n* = 2475) vs. foreign-born mothers (*n* = 592). Analyses were adjusted for country because of differences in follow-up rates. Foreign-born mothers were more often reported to have low education, be single, multiparous, and to have at least one unemployed parent in their household than native mothers; their children had lower rates of BPD and higher average SDQ total scores. Table 2 outlines children’s average LDs between their L1 to the host country’s official language by native vs. foreign-born mothers per each participating country. For detailed information on the distribution of languages across countries please see Appendix 1.

**Table 1:** Descriptive characteristics of the EPICE-SHIPS sample assessed at both 2 and 5 years by native vs. foreign-born mothers (*N* = 3067).

	Mother’s country of birth		
	Native *(n* = 2475*)*	Foreign *(n* = 592*)*	*p*-value^a^
Infant gestational age (*Mean (SD*), weeks)	28.82 (2.01)	28.79 (2.03)	0.562
Mother’s age (*Mean (SD*), years)	31.52 (5.72)	31.98 (5.64)	0.103
Mother’s educational level (*n (%))*			<0.001
Low	352 (14.3)	144 (24.4)	
Medium-High	2103 (85.7)	447 (75.6)	
Single mother status (*n (%))*			0.043
Yes	274 (11.1)	83 (14.2)	
No	2188 (88.9)	502 (85.8)	
Household employment (*n (%))*			<0.001
At least one parent unemployed	228 (9.3)	106 (18.1)	
Employed	2226 (90.7)	479 (81.9)	
Type of pregnancy (*n (%))*			0.700
Singleton	1681 (67.9)	402 (67.9)	
Multiple	794 (32.1)	190 (32.1)	
Parity (*n (%))*			<0.001
Primiparous	1550 (63.2)	317 (54.0)	
Multiparous	902 (36.8)	270 (46.0)	
Infant sex (*n (%))*			0.188
Female	1141 (46.1)	291 (49.2)	
Male	1334 (53.9)	301 (50.8)	
Severe neonatal morbidity^b^ (*n (%))*			0.375
Yes	255 (10.5)	47 (8.2)	
No	2178 (89.5)	527 (91.8)	
BPD^c^ (*n (%*))			0.041
Yes	352 (14.3)	59 (10.1)	
No	2103 (85.7)	524 (89.9)	
SDQ total score at 5 years *(Mean (SD*))	9.41 (5.69)	10.06 (5.74)	0.007

*SD* standard deviation, *SDQ* Strengths and Difficulties Questionnaire.

Please note: ^a^adjusted for children’s country of birth;

^b^Severe neonatal morbidity defined as a composite measure of cystic periventricular leukomalacia, intraventricular haemorrhage grades III or IV, severe necrotizing enterocolitis requiring surgery or peritoneal drainage, or retinopathy of prematurity at least stage 3;

^c^*BPD* Bronchopulmonary dysplasia with supplemental oxygen and/or ventilatory support (continuous positive airway pressure or mechanical ventilation) at 36 weeks of postmenstrual age.

**Table 2:** Children’s average linguistic distance (LD) between their L1 to the host country’s official language by native vs. foreign-born mothers per each participating country (*N* = 3067).

		Mothers’ country of birth	
		Native	Foreign-born
**Children’s country of birth**			
Belgium	*n*	150	25
	LD *Mdn*; *M (SD)*	0.00; 3.78 (18.59)	0.00; 41.56 (47.91)
Denmark	*n*	112	8
	LD *Mdn*; *M (SD)*	0.00; 0.00 (0.00)	0.00; 0.00 (0.00)
Estonia	*n*	120	9
	LD *Mdn*; *M (SD)*	0.00; 23.17 (42.14)	99.29; 66.19 (49.65)
France	*n*	459	212
	LD *Mdn*; *M (SD)*	0.00; 0.00 (0.00)	0.00; 6.27 (23.66)
Germany	*n*	169	66
	LD *Mdn*; *M (SD)*	0.00; 4.31 (19.52)	0.00; 43.15 (47.71)
Italy	*n*	452	120
	LD *Mdn*; *M (SD)*	0.00; 0.13 (2.69)	0.00; 25.66 (39.26)
The Netherlands	*n*	114	13
	LD *Mdn*; *M (SD)*	0.00; 0.85 (9.10)	0.00; 15.32 (37.40)
Poland	*n*	172	–
	LD *Mdn*; *M (SD)*	0.00; 0.00 (0.00)	–
Portugal	*n*	348	77
	LD *Mdn*; *M (SD)*	0.00; 0.23 (4.22)	0.00; 12.43 (30.98)
Sweden	*n*	93	25
	LD *Mdn*; *M (SD)*	0.00; 1.05 (10.11)	88.22; 63.91 (42.51)
United Kingdom	*n*	286	37
	LD *Mdn*; *M (SD)*	0.00; 1.01 (9.81)	0.00; 46.73 (48.70)

*LD* linguistic distance, *Mdn* median, *M* mean, *SD* standard deviation.

Please note: an LD of 0 indicates that the family L1 is the same as the host country’s official language, higher LD values indicate a larger distance.

Unadjusted models showed that higher LD and mothers’ foreign country of birth were each associated with higher SDQ total scores (Table 3, Model 1). When both LD and immigrant status were included in the model simultaneously (Model 2), only LD remained significant (0.02 [0.01, 0.03]), with a 1-point higher LD corresponding to 0.02 points higher SDQ scores. Random effects additionally indicated substantial variations in the associations of LD and immigrant status with SDQ total scores according to families (5.17 [4.83, 5.53]) and countries of residence (1.32 [0.96, 1.82]). LD from children’s L1 to the country’s official language was independently associated with SDQ total scores when adjusting for social adversities, i.e., mothers’ education, single mother status, and household unemployment in Model 3 (0.02 [0.01, 0.03]).

**Table 3:** Multilevel linear mixed-effects models showing associations of VP children’s LD with SDQ total scores at age 5 years.

*Dependent variable:*	SDQ total score			
	Model 1 *(N* = 3067)	Model 2 *(N* = 3067)	Model 3 *(N* = 3020)	Model 4 *(N* = 2915)
***Fixed effects, coefficient (95% confidence interval)***				
LD	0.02 (0.02, 0.03)***	0.02 (0.01, 0.03)***	0.02 (0.01, 0.03)***	0.02 (0.01, 0.03)***
Mother foreign country of birth	0.63 (0.16, 1.10)**	0.40 (−0.26, 1.07)	0.15 (−0.61, 0.92)	0.24 (−0.44, 0.92)
Mother’s education (high vs. low)			−1.45 (−2.26, −0.64)***	−1.43 (−2.27, −0.59)***
Single mother			−0.62 (−1.28, 0.04)	−0.60 (−1.14, −0.06)*
Household unemployment			1.73 (0.57, 2.90)**	1.45 (0.47, 2.42)**
Gestational age (weeks)				−0.08 (−0.19, 0.04)
Biological sex (female vs. male)				−1.53 (−2.01, −1.05)***
Multiple birth				−0.94 (−1.32, −0.56)***
Parity (multi- vs. primiparous)				−0.59 (−1.02, −0.16)**
Severe neonatal morbidity^a^				1.25 (0.32, 2.18)**
BPD^b^				1.01 (−0.34, 2.37)
Mother’s age (years)				−0.05 (−0.07, −0.02)**
constant		8.88 (7.54, 10.22)***	10.79 (8.64, 12.94)***	15.11 (11.53, 18.70)***
***Random effects by family***				
SD (constant)		5.17 (4.83, 5.53)*	5.09 (4.75, 5.45)*	4.97 (4.63, 5.33)*
***Random effects by country***				
SD (constant)		1.32 (0.96, 1.82)*	2.15 (1.83, 2.53)*	1.24 (0.86, 1.79)*
***Log-pseudolikelihood***		−14,827.37	−14,568.20	−13,852.13

*LD* linguistic distance, *VP* very preterm, *SDQ* Strengths and Difficulties Questionnaire, *SD* standard deviation, *BPD* bronchopulmonary dysplasia. Model 1 = unadjusted; Model 2 = LD and immigrant status adjusted for each other; Model 3 = additionally adjusted for mothers’ education, single mother status, household unemployment; Model 4 = fully adjusted, including LD, immigrant status, mothers’ education, single mother status, household unemployment, child GA, sex, multiple birth, mothers’ age, parity, BPD, severe neonatal morbidity; all models adjusted for IPW; for fixed effects *p < 0.05; **p ≤ 0.01; ***p ≤ 0.001; for random effects * marks a 95% confidence interval not including 0; significant effects of interest marked in **bold;**

^**a**^Severe neonatal morbidity defined as a composite measure of cystic periventricular leukomalacia, intraventricular haemorrhage grades III or IV, severe necrotizing enterocolitis requiring surgery or peritoneal drainage, or retinopathy of prematurity at least stage 3;

^b^BPD = Bronchopulmonary dysplasia with supplemental oxygen and/or ventilatory support (continuous positive airway pressure or mechanical ventilation) at 36 weeks of postmenstrual age.

In the fully adjusted Model 4, additionally including child GA, sex, multiple births, mothers’ age, parity, BPD, and severe neonatal morbidities, fixed effects of children’s LDs from their L1 to the host country’s official language remained stable (0.02 [0.01, 0.03]). As before, random effects indicated substantial variations in the associations between children’s LD and SDQ total scores according to families and countries of residence. In this model, if all other factors were held stable, a 10-point higher LD would result in a 0.2-point higher SDQ total score.

Analyses were repeated within the subgroup of children with foreign-born mothers (Table 4). As in the full sample, the fixed effect of immigrant children’s LDs on SDQ total scores remained stable across all models (0.02 [0.01, 0.02]).

**Table 4:** Multilevel linear mixed-effects models showing associations of VP immigrant children’s LD with SDQ total scores at age 5 years.

*Dependent variable:*	SDQ total score		
	Model 1 (*n* = 428)	Model 2 (*n* = 423)	Model 3 (*n* = 399)
***Fixed effects, coefficient (95% confidence interval)***			
LD	0.01 (0.00, 0.03)***	0.02 (0.02, 0.03)***	0.02 (0.01, 0.02)***
Mother’s education (high vs. low)		−1.98 (−2.64, −1.31)***	−1.86 (−2.60, −1.13)***
Single mother		−0.17 (−1.25, 0.91)	−0.32 (−0.96, 0.32)
Household unemployment		0.51 (−0.49, 1.51)	0.14 (−0.82, 1.11)
Gestational age (weeks)			−0.36 (−0.58, −0.14)**
Biological sex (female vs. male)			−1.11 (−2.47, 0.25)
Multiple birth			−1.37 (−2.42, −0.33)**
Parity (multi- vs. primiparous)			−0.98 (−1.88, −0.08)*
Severe neonatal morbidity^a^			2.04 (−3.73, 7.81)
BPD^b^			−1.99 (−5.17, 1.18)
Mother’s age			−0.00 (−0.06, 0.06)
constant	9.05 (7.91, 10.19)***	12.35 (10.54, 14.16)***	25.28 (18.20, 32.35)***
***Random effects by family***			
SD (constant)	5.14 (4.75, 5.56)*	5.07 (4.63, 5.55)*	4.83 (4.48, 5.19)*
***Random effects by country***			
SD (constant)	1.31 (0.73, 2.34)*	1.38 (0.77, 2.45)*	1.49 (0.90, 2.47)*
***Log-pseudolikelihood***	−2315.94	−2285.64	−2,120.70

*LD* linguistic distance, *VP* very preterm, *SDQ* Strengths and Difficulties Questionnaire, *SD* standard deviation, *BPD* bronchopulmonary dysplasia. Model 1 unadjusted; Model 2 adjusted for mothers’ education, single mother status, household unemployment; Model 4 fully adjusted, including LD, mothers’ education, single mother status, household unemployment, child GA, sex, multiple birth, mothers’ age, parity, BPD, and severe neonatal morbidity; all models adjusted for IPW; for fixed effects *p < 0.05; **p ≤ 0.01; ***p ≤ 0.001; for random effects * marks a 95% confidence interval not including 0; significant effects of interest marked in **bold;**

^a^Severe neonatal morbidity defined as a composite measure of cystic periventricular leukomalacia, intraventricular haemorrhage grades III or IV, severe necrotizing enterocolitis requiring surgery or peritoneal drainage, or retinopathy of prematurity at least stage 3;

^b^*BPD* Bronchopulmonary dysplasia with supplemental oxygen and/or ventilatory support (continuous positive airway pressure or mechanical ventilation) at 36 weeks of postmenstrual age.

## Discussion

This study, for the first time, documents associations of LD between VP children’s L1 and host countries’ official languages with higher behavioural and socio-emotional problems at 5 years of age. Our findings from the EU-wide EPICE-SHIPS cohort of children born VP show that there is also an association of immigrant status with higher behavioural and socio-emotional problems, however its effect is not independent of LD. Importantly, the contribution of LD to explaining children’s behavioural and socio-emotional problems remained stable even after adjusting for a wide range of social, biological, and perinatal clinical factors. This points to the critically important role of language barriers for the social and emotional development of immigrant children born VP.

It is important to note that the size of the fixed effect of LD (i.e., 0.02 [0.01, 0.03]) in our models may seem small in comparison to some other control variable effects such as GA (i.e., −0.08 [−0.19, 0.04]) for example. However, these coefficients represent the change in the mean response (SDQ total score) associated with a 1-unit change in that term. Accordingly, for example, if all other factors in the model would be held stable, among Romanian-speaking immigrant children living in Italy whose LD is 55.78 (*n* = 13 in the current sample) this would translate into an average of 1.12 more points on the SDQ total score compared with native Italian children, whereas the SDQ total score would be on average 2.00 points higher among Turkish-speaking immigrant children living in Germany (LD = 99.77, *n* = 9) compared with native German children. Considering the overall range and distribution of SDQ total scores in this European sample of VP children, a 2-point difference is more than 1/3 of a standard deviation. For those readers who prefer comparing standardised coefficients despite their limitations in mixed-effects models, the following comparison may be helpful: In the fully adjusted Model 4 in the total sample, standardised fixed effects of the continuous variables in the model were LD = 0.43 [0.17, 0.68], gestation = −0.22 [−0.55, 0.11], and mother’s age = −0.28 [−0.45, −0.10], respectively.

The finding that the association of immigrant status when operationalised as a binary category, i.e., native vs. foreign-born mother, with children’s behavioural and socio-emotional problems was attenuated when the continuous LD score was introduced to the model points to the importance of assessing language barriers when studying immigrant children’s developmental outcomes.^15,17,34^ There are wide variations between immigrants’ experiences depending on a range of intersectional factors including their heritage language, country of origin, reasons for migration, legal status, ethnicity, educational qualifications, and economic resources,^19,26,32^ as well as host countries’ immigration policies and societies’ willingness to integrate immigrant populations.^59^ The traditionally used oversimplification of the variable ‘immigrant’ is often masking these heterogeneous conditions shaping immigrants’ lived experiences. The operationalisation of language barriers via LD helps unveil important aspects of the equation.^35^ Host country official language skills constitute a critical resource for immigrant families when it comes to navigating their new society,^12^ since language difficulties create barriers, misunderstandings, stigma, inequality, and discrimination. Proficiency in a country’s majority language correlates with migrant women’s prenatal care utilisation,^15–17^ self-perceived prenatal care communication quality,^60^ overall health.^18^ Higher levels of behavioural and socio-emotional problems among VP children growing up with language barriers are likely an indirect consequence of their mothers’ and fathers’ lived experiences in navigating the host society and its social systems. Immigrant families’ heritage languages constitute a core part of their identity and children’s multilingual skills warrant support,^21,61^ especially in today’s rapidly changing diverse societies. Language and communication with others are crucial for children’s socio-emotional development. In infancy, mothers and fathers are the most important environmental agents for their children’s socialization, but as children grow older and participate in other daily contexts such as preschool and peer groups, they need to understand and communicate more with other relevant people including teachers, friends, neighbours, etc. If children grow up with a high LD between their L1 and the language of their out-of-home environment, then communication may be compromised, which may cascade into feelings of frustration or isolation, and subsequently increased risk for behavioural and socio-emotional problems. Our findings of the stable association between children’s LD and their behavioural and socio-emotional problems emphasize the importance of better accounting for linguistic heterogeneity in research, policy, and practice with immigrant populations. It is critical that future studies replicate the associations in other samples, across other developmental dimensions, and also employ mixed-methods designs as well as information collected from representatives of the host population (e.g., children’s teachers, paediatricians, school psychologists) to better understand the complex and dynamic mechanisms at play here.

As expected, social, biological, and perinatal clinical factors made important contributions to children’s behavioural and socio-emotional problems, including mothers’ education, single mother status, and household unemployment as well as child sex, multiple birth, mother’s age, parity, and severe neonatal morbidities. There is substantial overlap among these vulnerabilities, creating intersectionality and multiple combined risks for certain individuals.^26^ In particular among immigrants, it is important to stress the contribution of mother’s level of education to their children’s behavioural and socio-emotional problems.^24^ For instance, if all other factors in the model were held stable, children of immigrant mothers with medium-high educational qualifications would have an average of 1.86 lower points on the SDQ total score compared with children of immigrant mothers with low education. While families’ heritage languages and mothers’ educational attainment are not easily changed through intervention, they represent valid and reliable markers that can be used as easy-to-implement screening tools for research, policy, and practice, and open new avenues to intervention.

### Strengths and limitations

The main strength of this population-based, prospective cohort study is the large sample size of children born VP across 11 European countries. As in most longitudinal studies, loss to follow-up may have biased our findings, especially considering that participant attrition at 5 years was associated with social disadvantage (Aubert et al.,^57^). It was not in the scope of this study to provide a detailed analysis of the reasons for dropout, especially because the main predictor of interest (home language use) was collected at the 2-year follow-up assessment and not at birth. However, IPW was used in all models to correct for possible biases associated with selective participation at 2 and 5 years. The operationalisation of language barriers in the form of LD across a wide, diverse range of immigrant families living in different European contexts allowed us to break up the classic but oversimplified categorisation of participants into “native vs. foreign-born”. At the same time, the continuous LD score provides an elegant solution to assess variations in language barriers, with minimal resource requirements for data collection. In fact, most studies and data registries contain information on L1, or at least on population members’ countries of birth, therefore allowing the implementation of screening for LD. We are hopeful that future studies will use this tool to replicate our findings, and that policymakers may consider a wide implementation, for example, to facilitate decisions about the provision of language learning resources to new immigrants.

This study also has weaknesses. Despite its continuous scoring, LD was not normally distributed across the population, and unequally across countries. We used mixed-effects models to account for the residual data structure, i.e., different distributions between countries, and to minimise the risk for false estimations. Accordingly, the stability of the LD coefficients across all models and within the foreign-born subsample, along with stable overall fit values indicated robust findings. The SDQ was administered to participating parents in each country’s official language, but not in immigrants’ L1s. This has very likely created participation bias (due to mothers dropping out whose host country language skills were not sufficient) as well as response bias (due to misunderstandings of instructions and item content). In addition, depending on their socio-cultural backgrounds, immigrant parents may tend to perceive child behaviours differently than native parents.^62,63^ However, we adjusted for social risk factors and still found a stable effect of LD. Future studies should ensure that all assessments are administered in participants’ L1s,^64^ and to select standardised instruments that are as culturally fair as possible.^65^ We did not correct for the length of stay of the mother in the host country, although this might play a role in the degree of experienced language barriers. Independent variable information was collected at birth, two, and five years of child age, and for some variables such as maternal level of education information from one timepoint was supplemented with another if there were missing values. We did not account for possible intra-individual changes in demographic characteristics over time. Moreover, fathers also play an important role for their children’s development.^66,67^ However, for the current study, the binary immigrant variable was operationalised based on mothers’ country of birth as the main exposure. It was not possible to include detailed data on whether fathers were foreign-born due to missing data. As a result, we may have misclassified children with immigrant fathers as ‘native’, potentially underestimating the effect of immigrant status on SDQ scores. The LD variable however was operationalised based on children’s L1s, including languages spoken by fathers.

Finally, the limited available literature points to a possibly dynamic and interactive developmental double jeopardy of language barriers and VP birth.^48,68^ However, this hypothesis could not be assessed with the current sample as all children were born VP. Future studies should plan with a continuous gestational age or 2 × 2 group design to address this question and also assess the size of the association between LD and developmental outcomes across different gestational age groups.

### Conclusion

A larger LD between VP children’s L1 and countries’ official languages is associated with higher behavioural and socio-emotional problems at 5 years of age. Language barriers play a critically important role for the development of immigrant children born VP. Researchers, practitioners, and policymakers may consider implementation of screening for LD as part of regular follow-up after VP birth.

## Supplementary information

Appendix 1

## Data Availability

The datasets generated during and/or analysed during the current study are not publicly available due to reasons of confidentiality and participant personal data privacy, but aggregated data are available from the corresponding author on reasonable request.
